# Performance of the Eversense versus the Free Style Libre Flash glucose monitor during exercise and normal daily activities in subjects with type 1 diabetes mellitus

**DOI:** 10.1136/bmjdrc-2020-001193

**Published:** 2020-08-11

**Authors:** Marion Fokkert, Peter R van Dijk, Mireille A Edens, Alberto Díez Hernández, Robbert Slingerland, Rijk Gans, Elías Delgado Álvarez, Henk Bilo

**Affiliations:** 1 Department of Clinical Chemistry, Isala, Zwolle, NA, The Netherlands; 2 Internal Medicine, University of Groningen, University Medical Center Groningen, Groningen, The Netherlands; 3 Diabetes Research Center, Isala, Zwolle, NA, The Netherlands; 4 Department Innovation and Science, Isala, Zwolle, NA, The Netherlands; 5 Sección de Endocrinología, Hospital del Bierzo, Ponferrada, Castilla y León, Spain; 6 Sección de Diabetes, Universidad de Oviedo, Oviedo, Asturias, Spain; 7 Sección de Diabetes, Hospital Universitario Central de Asturias, Oviedo, Asturias, Spain

**Keywords:** blood glucose monitoring, exercise, continous blood glucose monitor(s)

## Abstract

**Introduction:**

Accurate blood glucose measurements are important in persons with diabetes during normal daily activities (NDA), even more so during exercise. We aimed to investigate the performance of fluorescence sensor-based and glucose oxidase-based interstitial glucose measurement during (intensive) exercise and NDA.

**Research design and methods:**

Prospective, observational study in 23 persons with type 1 diabetes when mountain biking for 6 days, followed by 6 days of NDA. Readings of the Eversense (fluorescence-based continuous glucose monitoring (CGM); subcutaneously implanted) and of the Free Style Libre (FSL; glucose oxidase-based flash glucose monitoring (FGM); transcutaneously placed) were compared with capillary glucose levels (Free Style Libre Precision NeoPro strip (FSLCstrip)).

**Results:**

Mean average differences (MAD) and mean average relative differences (MARD) were significantly different when comparing exercise with NDA (reference FSLCstrip); Eversense MAD 25±19 vs 17±6 mg/dL (p<0.001); MARD 17±6 vs 13%±6% (p<0.01) and FSL MAD 32±17 vs 18±8 mg/dL (p<0.01); MARD 20±7 vs 12%±5% (p<0.001).

When analyzing the data according to the Integrated Continuous Glucose Monitoring Approvals (class II–510(K) guidelines), the overall performance of interstitial glucose readings within 20% of the FSLCstrip during exercise compared with NDA was 69% vs 81% for the Eversense and 59% vs 83% for the FSL, respectively. Within 15% of the FSLCstrip was 59% vs 70% for the Eversense and 46% vs 71% for the FSL.

**Conclusions:**

During exercise, both fluorescence and glucose oxidase-based interstitial glucose measurements (using Eversense and FSL sensors) were less accurate compared with measurements during NDA. Even when acknowledging the beneficial effects of CGM or FGM, users should be aware of the risk of diminished accuracy of interstitial glucose readings during (intensive) exercise.

**Significance of this study**

**What is already known about this subject?**

- Despite reasonable good accuracy of interstitial glucose registrations under standardized conditions, interstitial glucose readings during exercise do tend to be less accurate.
- Qualitatively comparable glucose results of real-time continuous glucose monitoring and flash glucose monitoring show differences in accuracy.

**What are the new findings?**

- During exercise, interstitial glucose readings indeed are less accurate compared with readings during normal daily life activities, often with clinically relevant differences, compared with capillary measurements.

**How might these results change the focus of research or clinical practice?**

- Persons with diabetes mellitus and healthcare providers should be aware of the pitfalls when interpreting glucose interstitial readings during exercise, and should be given proper education with regard to the described inaccuracy.

## Introduction

Regular exercise by persons with diabetes mellitus (DM) contributes to physiological and health benefits on the short and long term.^1–3^ Maintaining acceptable glucose concentrations during sports activities is challenging, however. The more intense the sport activity and the more extreme the conditions, the larger the challenge of balancing energy intake and insulin use. Therefore, particularly during exercise, timely and accurate blood glucose readings are of importance to enhance chances on good metabolic control, allowing maximal performance.

During strenuous circumstances, an alternative to capillary self-measurement of blood glucose (SMBG) levels performed is interstitial glucose measurements. At present, real-time continuous glucose monitoring (rt-CGM) and flash glucose monitoring (FGM) are available as methods for interstitial glucose measurements. If used appropriately, with rt-CGM the user often only needs to calibrate the sensor at some time points daily and being able to act and react on alarms preset at certain cut-off points. In contrast, FGM users use a factory-calibrated sensor, which with the presently available version in The Netherlands needs scanning for obtaining the required information.^4^

Some authors report qualitatively comparable glucose results of rt-CGM and FGM,^5^ although others observed differences in accuracy.^6^ However, these comparative studies are virtually always done under routine or standardized clinical circumstances. With such standardized testing, it can be assumed that factors such as interstitial fluid dynamics and interstitial glucose concentrations will behave more or less in a stable pattern during the testing periods. In contrast, real-life (intensive) exercise most likely will lead to more frequent and profound changes in interstitial fluid dynamics and thus in interstitial glucose concentration.^7^ Therefore, performance of interstitial glucose measurements might be quite different during intensive exercise conditions as compared with a performance during normal daily activities (NDA). Furthermore, depending on the technique used to measure interstitial glucose concentrations, the change in interstitial fluid dynamics might also affect reading outcomes differently.

In an earlier study, we found that during NDA and standardized tests FGM reading showed lower glucose concentrations than actually present with capillary measurements in the lower ranges, and higher than found in the higher ranges.^8^ In contrast, FGM performance proved to be different during intensive exercise in a real-life setting, showing comparatively higher glucose readings than actually present, but also less accuracy throughout the whole measurement spectrum.^9^ The rt-CGM performance during exercise in that study also was less accurate than hoped for. Both the FGM and rt-CGM devices used in that study used a glucose oxidase based method for measuring glucose.^10 11^

Recently, an implantable rt-CGM device has been introduced (Eversense), using fluorescence-based glucose measurement technique,^12^ with an accuracy comparable to other rt-CGM devices.^13^ The Eversense is an implantable rt-CGM device, needs calibrating twice daily, and ideally functions for 6 months. The Free Style Libre (FSL) FGM system has a transcutaneous probe, is factory calibrated and functions for 14 days.

Given the importance of reliable glucose measurements during intensive exercise, we aimed to compare in a real-life setting the accuracy of two interstitial devices that use both a different method (glucose oxidase based vs fluorescence based) to measure glucose in persons with type 1 diabetes mellitus (T1DM). Furthermore, since differences in accuracy may differ depending on the circumstances and activity level,^14^ measurements were also performed during a period of NDA with no sports activities in the week after the mountain biking.

## Research design and methods

### Study design

This was a prospective, observational study. The aim was to compare two different methods for interstitial glucose measurements: fluorescence based (using the Eversense) versus glucose oxidase based (using FSL) in two consecutive time periods, with first a 6-day period of intensive exercise, followed by a 6-day period of NDA.

Capillary glucose measurements were performed with Precision Neo Pro strips (Free Style Libre Capillary glucose measurement strip (FSLCstrip)).^8^ In earlier research, this capillary measurement strip was verified to be closely comparable with National Institute of Standards and Technology (NIST) standards to the gold reference method (y=0.974 x−0.294, correlation 0.995).^8 15^ Therefore, it was decided to use the results of the FSLCstrip as the reference values.

### Setting and participants

This study was performed during the Bas van de Goor Foundation ‘webike2changediabetes’ challenge in September 2018 and in the week after the challenge (NDA, no sports activities). The Bas van de Goor Foundation is a non-profit organization that aims to promote sports and exercise in persons with DM. During the current challenge, a combined team of 29 persons with 27 T1DM and 2 type 2 diabetes mellitus from Spain and The Netherlands mountain biked in the Sierra Nevada, over a total distance of 263 km and a variable amount of altitude meters spread over the days (in total minimal 4753 up to 11 000 m) https://bvdgf.org/evenementen/evenement/27041/webike2changediabetes-2018/. The maximum altitude reached was 3398 m,^16^ but only for a short period of time. All participants in the Bas van de Goor Foundation challenge ‘webike2changediabetes’ in September 2018 were asked to participate in the study.

### Procedures

Eventually, 23 participants with T1DM of total of the 27 participants agreed to be included in the study. After obtaining written informed consent, baseline characteristics were collected before and at start of the challenge. Eversense devices were implanted in the back of the upper arm, at one center in The Netherlands and several centers in Spain in the month of August 2018, according to the operating instructions, and by certified care professionals. FSL were inserted prior (>12 hours) to the challenge on the back of the opposite upper arm. The algorithm used to assess the readings of the Eversense was the same as in the Precise I study,^13^ (being the initial Eversense algorithm; since then, a new algorithm has been implemented) for the FSL the FSL’s first glucose known algorithm was used with the first available version of the FSL without the alarm functions as present in the FSL 2 version.

Participants were asked to perform a total of (at least) 7 SMBGs per day using FSLCstrip test strips with the blood glucose meter incorporated in the FSL reader. Participants scanned the glucose value with the FSL reader, performed a finger prick and finished with another scan with the FSL reader. The maximum time allowed between the two scans was 2 min. Participants were asked to perform extra SMBGs if necessary, for instance, when experiencing symptoms related to hypoglycemia. Eversense and FSL readings, taken within maximal 2 min of the capillary FLSCstrip measurement, were considered to be valid for comparison. For the Eversense, two FSLCstrip measurements per day were used for the calibration of the Eversense: these two calibration measurements were not included in the analysis comparing the readings of the both interstitial measuring devices and the FSLCstrip.

### Outcomes

Primary outcome of the present study was the accuracy of both methods for interstitial glucose measurements fluorescence based (Eversense) versus glucose oxidase (FSL) with the FLSCstrip during exercise and during NDA. Results related to accuracy were (primarily) assessed by calculating mean absolute differences (MAD), mean absolute relative differences (MARD). Since control frequency was different between participants, MAD and MARD were calculated per person, not per glucose measurement (ie, for each participant, MAD and MARD was calculated for each measurement, and the individual MADs and MARDs were translated into a mean MAD and MARD for each participant, both for the exercise period and the NDA period.

Secondary outcomes included the accuracy of the interstitial readers when assessed according to the cut-off points as formulated in the Integrated Continuous Glucose Monitoring Approvals (class II–510(K) guidelines) (ICGMA guidelines)^17^ and comparisons between exercise and NDA with the Parkes error grid analysis. For the latter purpose, Eversense, FSL and FSLCstrip readings in the two defined periods were fitted on the error grids as described by Parkes *et al*.^18^

### Statistical methods

Data were expressed as number (percentage), mean (SD) or median (IQR) for normally distributed and non-normally distributed data, respectively. Normality was examined with Q-Q plots.

Concerning measurements maximally 2 min apart, the MAD and MARD between the Eversence and FSL were compared. For comparison of the exercise condition and NDA (subsequent weeks), data were analyzed as summary measure, that is, the average MAD and MARD for each participant during both conditions was used for analysis. Data were tested using the paired t-test.

The comparison with the cut-off points as formulated in the ICGMA guidelines^17^ was purely descriptive.

For risk assessment of possible differences between interstitial-based glucose values versus FSLCstrip measurements, a Parkes error grid analysis, with percentage of measurements within consensus error grid zones, was performed. Values in zones A and B are deemed clinically acceptable, whereas those in zones C, D and E are considered potentially unsafe.

Where applicable, a significance level of 5% was used. Analyses were performed using SPSS V.25.0 (IBM SPSS Statistics, Armonk, New York, USA) and Microsoft Excel Analyse-It (2010).

## Results

Baseline characteristics of the 23 study participants with T1DM are shown in table 1.

**Table 1:** Baseline characteristics

	n=23
Age (years)	43 (18–56)
Female (%)	3 (13)
HbA1c (mmol/mol)	52 (36–76)
Diabetes duration (years)	17 (4–27)
MDI/CSII	14/9

Data are presented as n (%) or median (range).

CSII, Continuous Subcutaneous Insulin Infusion; HbA1c, hemoglobin A1c; MDI, Multiple Daily Injections.

When looking at the combined glucose measurement results performed according to protocol, in total 908 data sets were considered suitable for analysis for Eversense and FSL with FSLCstrip comparison in the exercise week, and 814 combined data sets suitable for analysis of the NDA week. The difference in scan results in both weeks between the two FSL scan readings performed before and after the FSLCstrip measurement was 0–2 mg/dL with a few (<1%) readings up to 5 mg/dL, indicating that during the vast majority of readings the situation was sufficiently stable to allow comparison with the capillary readings. For calculation, the readings of the Eversense and FSL that are closest to the FSLC in terms of time were used.

Table 2 shows the differences in glucose readings in total, and in the ranges ≤70, 71–180 and >180 mg/dL.

**Table 2:** Comparisons at various glucose concentrations of Eversense versus FSL versus FSLCstrip measurements as reference

Range (mg/dL)	N	FSLCstrip (1)	Eversense (2)	FSL (3)
Exercise				
≤70	61	58±10	59±15	73±17**
71–180	544	125±30	117±39**	142±43**
>180	303	241±53	222±59**	276±76**
Total	908	159±72	148±71**	182±88**
Normal daily activity				
≤70	49	59±9	68±26*	60±13
71–180	504	127±30	122±33**	128±39
>180	261	238±47	223±51**	240±57
Total	814	158±67	151±64**	160±73

2 vs 1 and 3 vs 1.

*P<0.05; **P<0.001.

FSL, Free Style Libre; FSLCstrip, Free Style Libre Precision NeoPro strip.

In general, Eversense readings were significantly lower both during exercise and NDA (except for the hypoglycemic ranges where small numbers of pairs were present (n=61 and n=49)), whereas the FSL readings were significantly higher during exercise, but not different from the FSLCstrip readings during NDA.

In table 3, results are shown of 22 of the 23 participants.

**Table 3:** MAD and MARD of the (n=22) Eversense and FSL versus FSLCstrip measurements; glucose in mg/dL; results in mean and SD; reference value FSLCstrip

	MAD Exercise (mg/dL)	MAD Normal daily activity (mg/dL)	MARD Exercise (%)	MARD Normal daily activity (%)
Eversense	25±10	17±6**	17±6	13±6*
FSL	32±17	18±8*	20±7	12±5**

Exercise versus normal daily activity.

*P<0.01; **p<0.001.

FSL, Free Style Libre; FSLCstrip, Free Style Libre Precision NeoPro strip; MAD, mean average differences; MARD, mean average relative differences.

In one participant, the FSL sensor came loose from the arm (impacted) during the NDA week, which precluded inclusion of this one patient in the analysis. MADs and MARDs of Eversense and FSL compared with FSLCstrip were significantly higher during exercise compared with NDA; Eversense: MAD 25±10 vs 17±6 mg/dL (p<0.001) and MARD 17±6 vs 13%±6% (p=0.004); FSL: MAD 32±17 vs 18±8 mg/dL (p=0.002) and MARD 20±7 vs 12%±5% (p<0.001).

When analyzing the data according to the ICGMA guidelines,^18^ performance during the exercise week does not reach the agreed on cut-off levels in either category of glucose levels or in total (table 4).

**Table 4:** Performance of the Eversense and FSL with FSLCstrip according to ICGMA guidelines, using FSLCstrip as reference

	Exercise (n=908)	Normal daily activity (n=814)	ICGMA: lower bound of one-sided 95% CI
**Eversense**			
Hypoglycemia (<70 mg/dL)	72% (44/61)	76% (37/49)	>85% within ±15 mg/dL
	97% (59/61)	90% (44/49)	>98% within ±40 mg/dL
Euglycemia (70–180 mg/dL)	56% (307/544)	69% (345/504)	>70% within ±15%
	92% (498/544)	96% (486/504)	>99% within ±40%
Hyperglycemia (>180 mg/dL)	68% (205/303)	75% (195/261)	>80% within ±15%
	95% (287/303)	98% (256/261)	>99% within ±40%
*Overall*	*69%* (*537/908*) *59%* (*628/908*)	*70%* (*565/814*) *81%* (*655/814*)	*Within ±15%* *>87% within ±20%*
**FSL**			
Hypoglycemia (<70 mg/dL)	61% (37/61)	78% (38/49)	>85% within ±15 mg/dL
	92% (56/61)	100% (49/49)	>98% within ±40 mg/dL
Euglycemia (70–180 mg/dL)	47% (255/544)	67% (337/504)	>70% within ±15%
	90% (490/544)	96% (483/504)	>99% within ±40%
Hyperglycemia (>180 mg/dL)	47% (142/303)	81% (212/261)	>80% within ±15%
	90% (273/303)	99% (258/261)	>99% within ±40%
*Overall*	*46%* (*420/908*) *59%* (*533/908*)	*71%* (*575/908* *83%* (*672/814*)	*Within ±15%* *>87% within ±20%*

FSL, Free Style Libre; FSLCstrip, Free Style Libre Precision NeoPro strip; ICGMA, Integrated Continuous Glucose Monitoring Approvals (class II–510(K) guidelines).

This failure to reach those levels is especially pronounced with the stricter cut-off levels. The situation improves when assessing performance during the NDA period.

**Figure 1:**
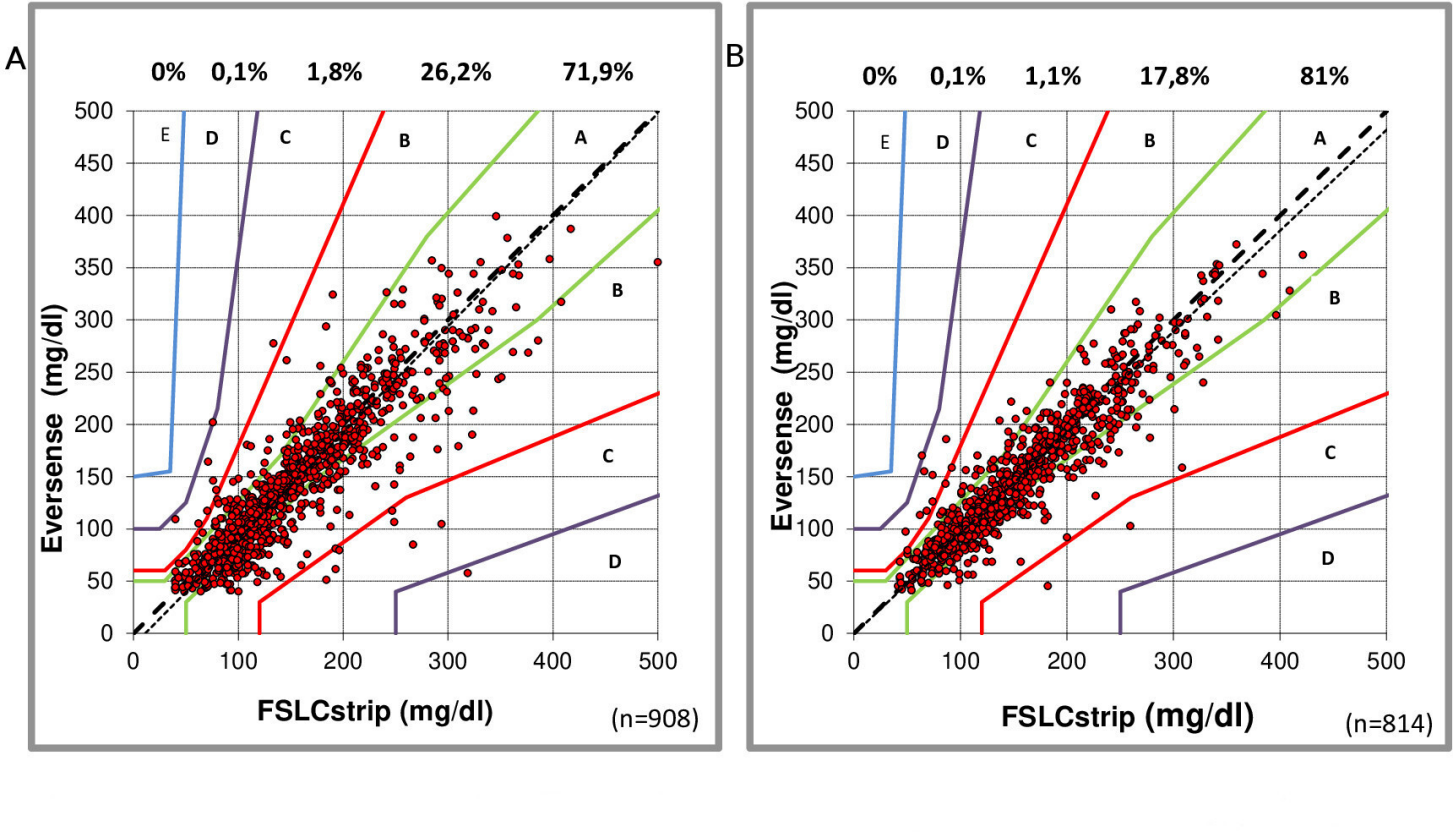
Comparison (Parkes error grids, ISO15197; 2013) between capillary measurements (Free Style Libre Precision NeoPro strip (FSLCstrip)) and Eversense readings during the exercise week (A) and normal daily activity week (B). Percentages of paired readings within error grid zones A, B, C, D and E are presented.

When assessing the performance of the Eversense CGM during exercise compared with the FSLCstrip, 98.3% of the comparisons fell within zones A and B (73.2% and 25.1%, respectively) of Parkes error grid analysis, with the slope following closely the x=y line: y=1.010 x−9.88, correlation 0.883 (figure 1A). In the NDA week, the Eversense versus FSLCstrip comparison showed 98.8% of the comparison within zones A and B (81.9% and 16.9%, respectively), again with the slope closely following the x=y line: y=0.96 x−0.11, correlation 0.920; figure 1B). Assessing the comparative readings during daily activities in zone B, the Eversense CGM tends to show slightly lower concentrations than measured by capillary assessment, this in contrast to the observations with the FSL.

**Figure 2:**
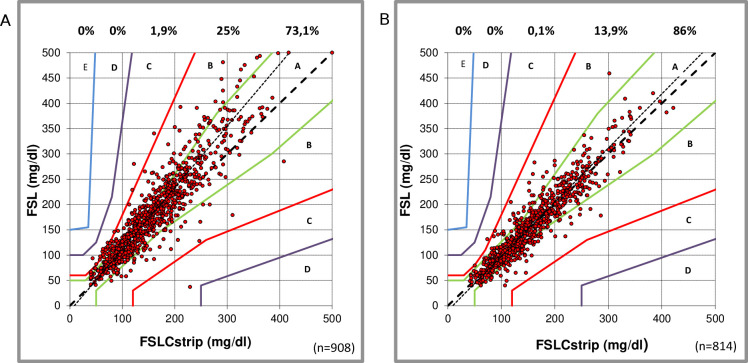
Comparison (Parkes error grids, ISO15197; 2013) between capillary measurements (Free Style Libre Precision NeoPro strip (FSLCstrip)) and Free Style Libre (FSL) readings during the exercise week (A) and normal daily activity week (B). Percentages of paired readings within error grid zones A, B, C, D and E are presented.

During exercise, only few comparative readings of FSL compared with FSLCstrip were outside the zones A and B, 98.2% (73.9% and 24.3%, respectively), with a majority (84%) of the B readings in the upper B region (figure 2A). The slope of the comparison line was 1.21, which allowed the conclusion, especially with higher glucose concentrations, that the flash FSL tends to show higher readings compared with capillary measurements (y=1.21 x−10.13, correlation 0.917). FSL measurements during NDA were well correlated to the FSLC measurements, with 99.9% of the points within the zones A and B (87.1% and 12.8%, respectively); the slope was 1.07 (y=1.07 x−9.57), with a correlation of 0.937; figure 2B).

## Discussion

In this study we demonstrated that when testing two different interstitial glucose measurement techniques, both the Eversense (fluorescence based) and FSL (glucose oxidase based) are less accurate during a period of exercise compared with a period of NDA. Importantly, differences are notable and relevant in virtually all categories. MAD and MARD during an exercise period are higher than during NDA, allowing the conclusion that both devices show less accuracy during exercise than during NDA. This conclusion is confirmed when measurements are assessed according to the ICGMA guidelines.

It has been shown that a reasonable or even high correlation between interstitial readings and capillary blood glucose concentrations is no guarantee for accuracy, and other measures such as MAD and MARD are needed for interpretation of the true accuracy.^5^ Even reporting overall MAD and MARD is an insufficient guarantee for adequate or even acceptable accuracy in the region that matters most to people with DM: the hypoglycemic range. Therefore, we advocate not restricting descriptions with regard to accuracy to a correlation (or correlations square) or overall MARD alone, and for comparison to adhere to the proposed ICGMA guidelines. Furthermore, even with rather high correlations as this study for example, for FSL during exercise (r=0.917; r²=0.841) and during daily activities (r=0.937; r²=0.878), there are considerable differences in MAD, MARD and performance according to the ICGMA guidelines. This again allows the conclusion that results of performance measurements need to be interpreted in context. Of course, first assessments need to be done under standardized conditions, but when interstitial measurement techniques are introduced and used in daily practice, probably (many) more factors need to be taken into account compared with venous and capillary blood measurements.

According to our findings, during periods of exercise the FSL tends to show higher than actual glucose concentrations, while the Eversense does show an opposite tendency. Whether these differences are caused by different algorithms translating the sensor signals into a glucose concentration value, or by another cause, is not known.

Taken together, this means that using interstitial glucose measurements during exercise is less accurate, which in its turn can have relevant consequences when inaccurate readings lead to wrong decisions. Users quickly learnt to handle the—sometimes—contradictory outcomes, often by assessing the trend information more than point measurements and controlling the readings through a capillary measurement when there was reason to doubt the result. Since we did not study these aspects, no formal conclusions can be drawn with regard to these aspects.

Providing a definitive explanation for the apparent different behavior of interstitial glucose sensors during NDA and during intensive exercise is complex. The more intensive the exercise, the more circulatory, tissue and interstitial changes will occur.^7^ Rate of glucose change and interstitial fluid flow will increase, possibly influencing the amount of glucose passing the FSL sensor per time unit. One possible explanation, at least partly, for the increased MARD during activity may be that the changes in interstitial readings lag behind the changes in blood glucose during prolonged aerobic exercise, which will contribute to an increase in MARD during exercise, as shown by Zaharieva *et al*.^19^

Others possible causes include outside and skin temperature, weather conditions, intensity of exercise and degree of insulation caused by clothing. Furthermore, since nobody will exercise 24 hours per day, periods of exercise will be alternated with periods of rest and less efforts during the exercise week. This also will have its influences.

Our study does have strengths and limitations. From a user’s point of view, testing in a real-life setting yields results, which often will be more applicable and recognizable than results obtained in a laboratory setting. Even if results during real life use are not as accurate as hoped for, studies like ours help to recognize the limitations and pitfalls of the use of rt-CGM and FGM under various circumstances.

One limitation of this study is our failure to ask the participants to exactly note down their actual time biking. Therefore, we could not perform a subanalysis of data acquired during the 24 hours of the exercise days during the exercise itself, and during the period(s)) outside the exercise hours. Speculating on whether that would have influenced the results, one should keep in mind that when discontinuing exercise, the interstitial situation will not immediately return to normal, nor will other possible influencing factors, such as insulin sensitivity. Furthermore, the influence of altitude is also unknown since we did not register altitude time over. As oxygen availability is 80% at 2000 m altitude and 75% at 2500 m altitude, this could have influenced the results.^20^ Theoretically, this difference in oxygen availability might influence interstitial oxygen availability, which in turn might affect glucose oxidation rate. During our study, time at altitude was limited; furthermore, one has to keep in mind that by mountain biking at altitude the primary demand will be to adjust breathing frequency and intensity according to the skeletal and cardiac muscle oxygen needs. Since this will be different for each individual, we felt it impossible to introduce a reliable correction for that factor. In addition, the influence of, among others, differences in environmental temperature, effects of altitude and concomitant decrease in partial oxygen pressure and humidity and participants’ hydration were not systematically assessed. Therefore, we could not correct for the possible influence of these factors. In a next study, we will need to combine FGM and rt-CGM registrations with additional devices to measure these factors. That would also allow a more detailed analysis of the influence of exercise proper, the resting periods during the exercise week (including sleeping) and a separate period of NDA.

Of notice, during the present study the first version of the FSL was used, thus missing the hypoglycemia alarm incorporated in the more recent FSL version.

Another major limitation is of course the use of a capillary glucose measurement using a glucose strip as the comparative method instead of, for example, a Yellow Springs Analyzer or glucose hexokinase measurement. In our opinion, the use of a Precision Neo Pro as ‘gold standard’ is justifiable, since this strip is aligned with NIST standards to the gold reference method.^16^ A further limitation is that the majority of the persons included in this study were male, possibly limiting the generalizability of our results.

## Conclusions

During exercise compared with daily life activities, interstitial glucose readings with both the Eversense (fluorescence based) and the FSL (glucose oxidase based) were less accurate, often with clinically relevant differences, compared with capillary measurements. This phenomenon was present throughout the whole range of glucose concentrations.

Even when acknowledging the limitations of a study in a real-life setting, users and healthcare providers should be aware of this and be given proper education with regard to the described phenomenon.

Although we did not formally test the described shortcomings during exercise, the vast majority of users considers using any form of CGM as a valuable tool for glucose monitoring, and gladly accept a lesser performance in exchange for more detailed information on glucose levels and glucose trends.
